# Supplementary material for: Intraoperative Visualization and Treatment of Salivary Gland Dysfunction in Sjögren’s Syndrome Patients Using Contrast-Enhanced Ultrasound Sialendoscopy (CEUSS)
Source: J Clin Med. 2023 Jun 20;12(12):4152. doi: 10.3390/jcm12124152 (PMC10298935; doi:10.3390/jcm12124152)
Supplement: Supplementary file 1 [file jcm-12-04152-s001.zip › jcm-2360340-supplementary.pptx]

## Slide 1
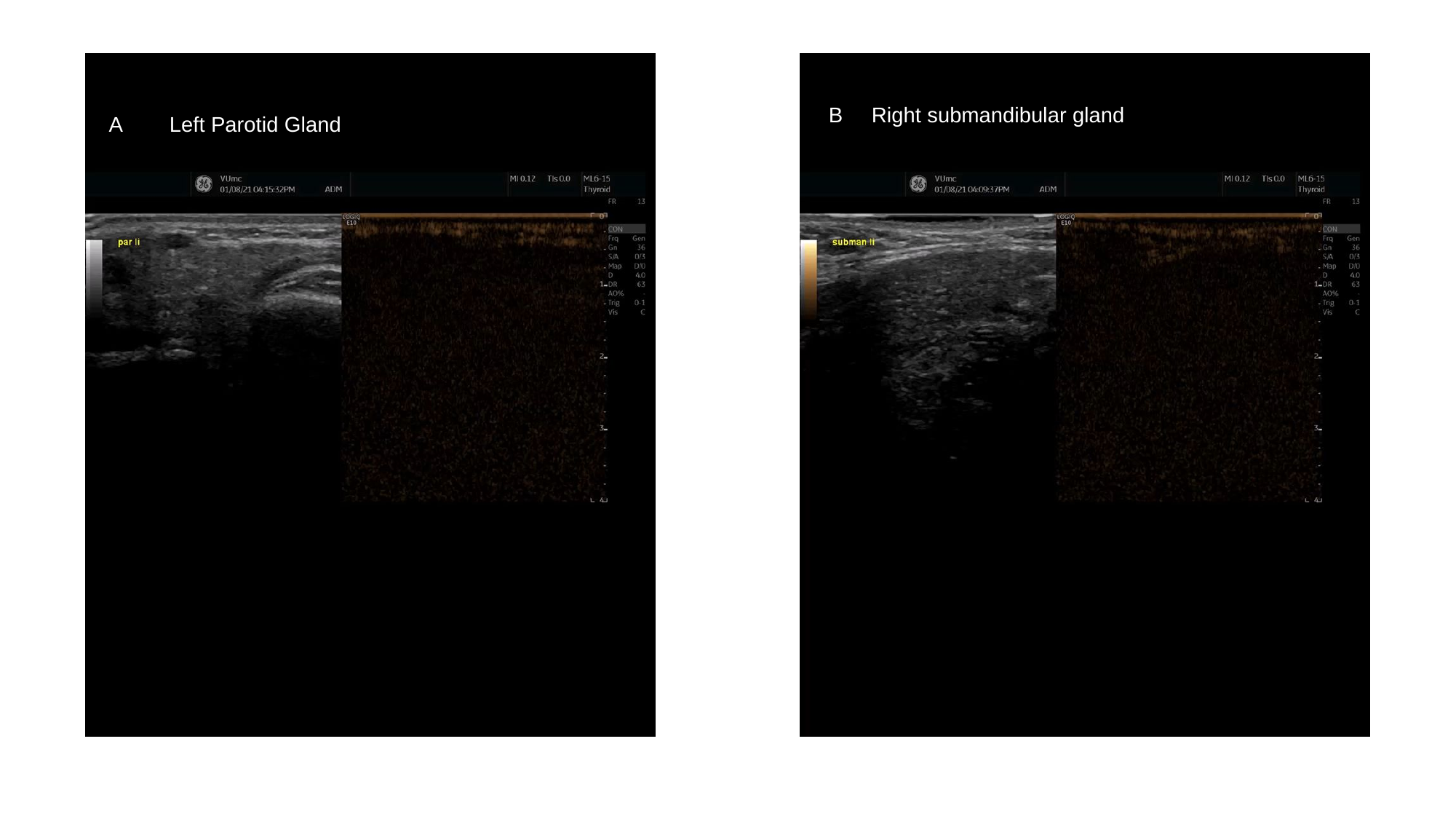

Right submandibular gland
B
A
Left Parotid Gland

## Slide 2
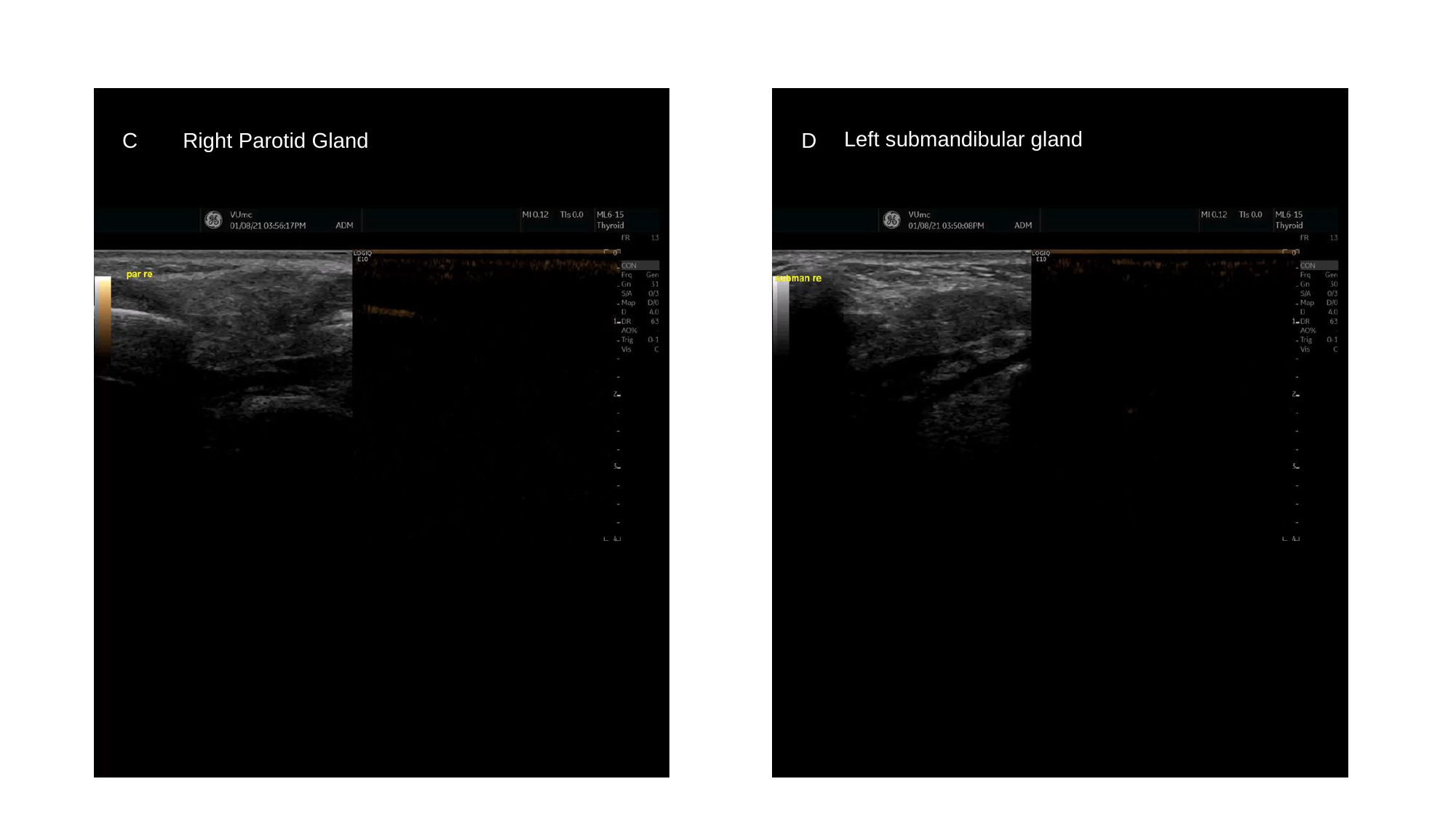

Left submandibular gland
Right Parotid Gland
D
C
